# Reported side-effects following Oxford/AstraZeneca COVID-19 vaccine in the north-west province, Iran: A cross-sectional study

**DOI:** 10.1371/journal.pone.0296669

**Published:** 2024-01-05

**Authors:** Majid Eterafi, Nasrin Fouladi, Majid Golizadeh, Hamidreza Shaker, Somaieh Matin, Elham Safarzadeh

**Affiliations:** 1 Students Research Committee, Ardabil University of Medical Sciences, Ardabil, Iran; 2 School of Medicine and Allied Medical Sciences, Ardabil University of Medical Sciences, Ardabil, Iran; 3 Social Determinants of Health Research Center, Ardabil University of Medical Sciences, Ardabil, Iran; 4 Cancer Immunology and Immunotherapy Research Center, Ardabil University of Medical Sciences, Ardabil, Iran; 5 Department of Internal Medicine, Emam Khomeini Hospital, Ardabil University of Medical Sciences, Ardabil, Iran; 6 Gastrointestinal and Liver Disease Research Center, Razi Hospital, Guilan University of Medical Sciences, Rasht, Iran; 7 Department of Microbiology, Parasitology, and Immunology, Ardabil University of Medical Sciences, Ardabil, Iran; Sheikh Hasina National Institute of Burn & Plastic Surgery, BANGLADESH

## Abstract

While the vaccination was introduced as a promising tool to control the Coronavirus disease 2019 (COVID-19) pandemic, concerns about vaccine-related side effects had grown. Due to the widespread administration of the COVID-19 vaccine worldwide for the first time, it was necessary to evaluate the safety and potential side effects in recipients. This study aims to assess, the incidence of adverse effects following Oxford-AstraZeneca vaccination and identify their related factors. In this cross-sectional survey-based study, 453 volunteers participated, including 235 men and 218 women. The reported adverse reactions from recipients of the ChAdOx1 nCoV-19 (Oxford-AstraZeneca) vaccine were collected by using a questionnaire. The findings showed that the incidence of adverse reactions, such as neurological, systematic, gastrointestinal, respiratory, and local symptoms were significantly higher after the first dose compared to the second dose. Systematic symptoms were the most prevalent reported side effects after the first and second dose injection. The demographical study of participants showed that individuals aged 18–34 and females were more prone to present adverse events following vaccination. However, no significant relationship was found between the occurrence of side effects and the recipients’ body mass index. Despite the life-saving role of vaccination against SARS-CoV-2, it may have some adverse reactions in recipients. The severity and frequency of side effects were different. So, they were dependent on several factors, including gender and age. Altogether, post-vaccination adverse reactions were mild and tolerable.

## 1. Introduction

SARS-CoV-2, a novel coronavirus, was identified as the cause of COVID-19. Due to the rapid increase in the number of infected cases, the World Health Organization (WHO) declared this outbreak a Public Health Emergency of International Concern (PHEIC) on January 30, 2020 [1, 2]. COVID-19 vaccine acceptance was low in some countries, despite the well-known fact that vaccines save lives. The main reason behind this hesitancy was the lack of information regarding vaccine safety and potential side effects. Moreover, the hasty approach with which vaccines under an emergency authorization, like the COVID-19 vaccines, are approved caused concern among some scientific community members [3].

On December 31, 2020, the mRNA vaccine "Pfizer BioNTech" and on February 15, 2021, the adenoviral vector vaccines ChAdOx1 nCoV-19 (Oxford-AstraZeneca) became the first two vaccines to be listed in the WHO Emergency Use Listing Procedure (EUL) for emergency use [4]. The ChAdOx1 nCoV-19 vaccine is a modified version of the chimpanzee adenovirus vaccine vector known as chAdOx1 which has a replication deficiency. It encodes the Spike protein antigen of the SARS-CoV-2 and is able to induce immune responses [5]. The Oxford-AstraZeneca vaccine also known by other names such as AZD1222 Vaccine, Covishield, and Vaxzevria was developed at the University of Oxford (Oxford, UK), Jenner Institute, in collaboration with AstraZeneca [6–8]. Preliminary results showed an efficacy of 70.4% for two doses of the vaccine and 64.1% protection against symptomatic disease after one dose. So these findings convinced some countries, such as the UK, to give emergency use authorization to adults over 18 years [5, 9]. In Iran, the permission for emergency administration of the AstraZeneca vaccine against COVID-19 was given on February 17, 2021 [10]. Following its use, several safety-related studies were planned. The results have shown that the vaccine has a good safety profile with a few self-limiting side effects. These include injection-site pain, headache, nausea, vomiting, diarrhea, swelling, redness at the injection site, dizziness, sleepiness, sweating, and abdominal pain. However, there have been rare instances of serious adverse effects reported, such as blood clots and anaphylaxis. [11]. Apart from the physiological aspect, psychological reactions such as Guillain-Barré syndrome, migraine headaches, paresthesia, delirium, hallucinations, and nervousness have been reported following vaccination [12].

Likewise, despite the evidence about the safety of the Oxford-AstraZeneca vaccine in primary studies, concerns about post-vaccination side effects grew after its general administration [13]. There is a need to gain more information on the potential side effects of vaccine injection and predisposing risk factors in recipients.

Therefore, the aim of this cross-sectional study was to evaluate the adverse events following the Oxford-AstraZeneca vaccination and examine the role of participants’ demographic and co-morbidity parameters in the incidence of vaccine-related side effects.

## 2.Materials and methods

### 2.1 Study design and participants

In the current cross-sectional survey-based study, we estimated the frequency of COVID-19 vaccine-related adverse events among recipients of the ChAdOx1 nCoV-19 (Oxford-AstraZeneca) vaccine between July 5, 2021 and November 14, 2021 in Ardabil province, Iran.

Vaccinated people in 150 urban health centers, rural health centers, and other ambulatory healthcare services were selected as the sampling framework. Then taking into account the conditions for entering the study, which included; receiving ChAdOx1 nCoV-19 (Oxford-AstraZeneca) vaccine, aged >18 years, not having passed more than two weeks since the first dose vaccination and consent to participate in the study, the study samples were selected and included in the study.

The sample size for this study was determined 453 individuals based on previous research [14] that took into account the occurrence rate of side effects ranging from 2% to 50%. Four hundred fifty-three individuals were randomly chosen from those who met the inclusion criteria, were classified by age and they were asked to complete the questionnaire through a phone call. The recruitment of individuals was as per ethical guidelines and that consent was obtained from each before enrolment. Prior to commencing the questionnaire, participants were provided the comprehensive information regarding the confidentiality of their personal data and the objectives of the research through a phone call and provided with a questionnaire link via social media sites if they expressed their willingness to take part in the study. Among these individuals, 5% had no interest in participating in the study, and 25% did not answer to phone calls. Individuals who were selected but had no interest in participating or could not be reached were randomly replaced by eligible respondents. Of note, if subjects had limited access to the internet and did not have the skills necessary to use it or other technical reasons, questioning was done through phone interview. Consent was obtained electronically from participants who agreed to enter the online survey. Prior to being presented with the survey in telephonic interviews, participants also provided oral consent.

### 2.2 Instrument

The questionnaire of this study consists of twenty-seven multiple-choice items created in the self-completed mode. Our survey included a list of adverse effects reported in the literature following COVID-19 vaccination [15–18], as well as opportunities for respondents to add additional side effects. We designed specific questions based on factors identified in the literature as associated factors with the development of the vaccine side effects, including age, gender, comorbidities, COVID-19 infection history, and number of vaccine doses received [17, 19]. The initial section of the survey comprised inquiries regarding demographic factors such as age, gender, blood group, Body Mass Index (BMI), and smoking habits. The second section of the survey comprised inquiries on comorbid conditions such as diabetes, hypertension, chronic respiratory ailments, cardiovascular diseases, cancer, and other chronic diseases. Additionally, this section encompassed an inquiry about the consumption of medication such as supplements and immunosuppressants. The third section of the survey asked about history COVID-19 infection and how severe it was. The fourth section focused on COVID-19 vaccination, including the number of doses received, as well as any side effects experienced.

The adverse effects were divided into five categories biased on experts: neurological, systemic, gastrointestinal, respiratory, and local. Systemic symptoms consisted of body pain, fever, shivering, fainting, postnasal drip, sore throat, and muscle spasms. Local side effects; injection site pain, injection site redness, injection site muscle spasm, and the neurological side effects included headache and confusion. Respiratory symptoms included coughing and shortness of breath. Gastrointestinal side effects included nausea, vomit, diarrhea, and loss of appetite (Anorexia).

A panel of a statistician, a medical immunologist, and an infectious diseases specialist were gathered to analyze the questionnaire draft and evaluate the validity of its content. The reliability of the questionnaire was checked before launching to a large group of participants by testing and retesting the questionnaire randomly on 30 vaccinated people and calculating Cronbach’s alpha. The overall reliability was 0.81, representing that the questionnaire tool was reliable.

The study was approved by the Research Ethics Committee of Ardabil University of Medical Sciences (IR.ARUMS.REC.1400.069). Before participation, each subject has given their informed agreement to participate online. No data were kept until the participant submitted their responses, and the participants were free to leave the study at any time without citing a reason.

### 2.3 Statistical analysis

Collected data was analyzed with SPSS (Statistical Package for Social Sciences) statistics for windows, version 21 (IBM Corp., Armonk, N.Y., USA). First, exploratory data analysis was performed to check the cleanliness of the data., Frequency and proportion were used to present the distribution of categorical variables for descriptive analysis while mean ± standard deviation (SD) or median (interquartile range, IQR) were used to express quantitative variables. Based on the distribution and the characteristics of the variables, parametric or non-parametric analytical statistics and Tukey’s post hoc test were used. The Monte Carlo and bootstrap tests, in cases of need, were also used. The multivariable logistic regression was performed to determine the factors associated with Oxford/AstraZeneca vaccine side effects. Odds ratios (ORs) with a 95% confidence interval (CI) were computed for different analyses. A p-value of 0.05 or less means it is significant.

## 3. Results

### 3.1 Demographic characteristics

This study analyzed the data of 453 participants who answered our survey, including 235 (51.9%) men and 218 (48.1%) women. All the participants received ChAdOx1 nCoV-19 (Oxford-AstraZeneca) vaccine. Of the participants,170 (37.5%) people received the first dose, and 283 (62.5%) received the both dose. Participants were divided into three age groups: 18–34 (N = 245, 54.1%), 35–64 (N = 118, 26.0%), and ≥65 (N = 90, 19.9%). The BMI of all the participants was calculated and sorted into four groups, including Underweight (N = 18, 4.0%), Normal (N = 184, 40.7%), Overweight (N = 181, 39.8%), and Obese (N = 70, 15.5%). Among all of the volunteers, 312 (69.0%) person had a medical history of underlying diseases, including cardiac (N = 32, 7.1%), respiratory (N = 13, 2.7%), diabetes (N = 38, 8.4%), blood pressure (N = 66, 14.6%), hepatic (N = 7, 1.3%), renal (N = 4, 0.9%), cancer (N = 5, 0.9%), and others (N = 48, 10.6%). Furthermore, 181 (39.9%) persons had a history of COVID-19. Detailed demographic information is shown in **Table 1**.

**Table 1:** Study subject’ demographics and general characteristics.

Variable	Category	N(%)
**Age**	18–34	245 (54.1)
	35–64	118 (26)
	≥65	90 (19.9)
**Gender**	Male	235 (51.9)
	Female	218 (48.1)
**BMI**	Underweight (<18.5)	18 (4)
	Normal (18.5–24.9)	184 (40.7)
	Overweight (25–29.9)	181 (39.8)
	Obese (>30)	70 (15.5)
**Blood type**	A	152 (33.6)
	B	77 (17)
	O	158 (34.9)
	AB	66 (14.6)
**Underlying diseases**	Yes	312 (69)
	No	141 (31)
	Diabetes	38 (8.4)
	Blood pressure	66 (14.6)
**Take supplements**	Yes	251 (55.4)
	No	202 (44.6)
**Take cortone immunosuppressive**	Yes	36 (7.9)
	No	417 (92.1)
**Smoke**	0	391 (86.3)
	1–10 (smoker)	42 (9.3)
	>10 (heavy smoker)	20 (4.4)
**Doses vaccine**	First one	170 (37.5)
	Both doses	283 (62.5)
**COVID-19 history**	Yes	181 (39.9)
	No	272 (60.1)
**Disease severity**	ICU	5 (2.7)
	Non-ICU	176 (97.3)

### 3.2 Reported adverse effects after AstraZeneca COVID-19 vaccine

Based on reports, the adverse effects after receiving the first dose occurred after 1.24 ± 0.536 days and prolonged for 2.95 ± 3.561 days. Similarly, the symptoms after the second dose injection were presented at 1.83 ± 1.06 days and persisted for 2.90 ± 5.06 days. According to the findings, the total number of reported symptoms after the first dose vaccination ranged from 0 to 19 (Mean = 5.98 ±3.45). Meanwhile, the number of reported adverse events after receiving the second dose ranged between 0 to 16 (Mean = 3.17±2.95). According to statistical analysis, the number of symptoms after the first dose, with a maximum of 19, was significantly higher than the number of side effects after the second dose (p-value of <0.0001). Reported adverse events were categorized into five groups: neurological, systematic, gastrointestinal, respiratory, and local. The occurrence of neurological, gastrointestinal, local, and systematic symptoms was significantly higher after the first dose in comparison to the second dose (p-value of <0.0001, <0.0001, <0.0001, <0.0001, respectively).

Findings showed that the systematic symptoms, including body pain, fever, shivering, fainting, nasal drip, sore throat, and muscular spasms, were frequently reported side effects after receiving the first and the second dose. Body pain was the most prevalent adverse event reported after receiving the first dose (N = 271, 59.8%). Likewise, body pain was the most reported symptom after the second dose injection (N = 84, 26.7%). In the second place, fainting was reported as a common side effect after vaccine injection after the first (N = 244, 53.9%) and second dose (N = 66, 21.0%). Additionally, fever as a systematic symptom (N = 241, 53.2%), headache that belongs to neurological symptoms (N = 207, 45.7%), and injection site pain, a local symptom (N = 182, 40.2%) were commonly reported adverse effects after the first dose. Also, fever (N = 62, 19.7%), injection site pain (N = 64, 20.3%), and headache (N = 51, 16.2%) were seen frequently after the second dose receiving. Reported adverse effects after vaccination are comprehensively presented in **Table 2.**

**Table 2:** Adverse events following first and second immunization of COVID-19 among the participants.

Variable	Category	Doses vaccine	
		First dose N(%)	Second dose N(%)
**Neurological symptoms**	**Headache**	207 (45.7)	51 (16.2)
	**Confusion**	97 (21.4)	14 (4.4)
**Systematic symptoms**	**Body pain**	271 (59.8)	84 (26.7)
	**Fever**	241 (53.2)	62 (19.7)
	**Shiver**	199 (43.9)	36 (11.4)
	**Faint**	244 (53.9)	66 (21)
	**Nasal drip**	22 (4.9)	4 (1.3)
	**Sore throat**	21 (4.6)	3 (1)
	**Muscular spasm**	61 (13.5)	8 (2.5)
**Gastrointestinal symptoms**	**Nausea**	57 (12.6)	12 (3.8)
	**Vomit**	25 (5.5)	9 (2.9)
	**Diarrhea**	27 (6)	6 (1.9)
	**No appetite (Anorexia)**	64 (14.1)	13 (4.1)
**Respiratory symptoms**	**Cough**	12 (2.6)	6 (1.9)
	**Shortness of breath**	20 (4.4)	4 (1.3)
**Local symptoms**	**Injection site pain**	182 (40.2)	64 (20.3)
	**Injection site redness**	31 (6.8)	7 (2.2)
	**Injection site Muscle spasm**	61 (13.5)	20 (6.3)
	**Injection site warmth**	54 (11.9)	7 (2.2)
	**Armpit pain**	13 (2.9)	2 (0.6)
	**Local itching**	8 (1.8)	3 (1)

### 3.3 The prevalence of side effects among participants with the underlying chronic disease after the first dose of AstraZeneca COVID-19 vaccine

The occurrence of adverse events after receiving the first dose in the participants with underlying chronic disease was investigated. A significant relationship (p-value = 0.006) between blood pressure and the incidence of post-vaccination side effects was observed (**S1 Table**). Findings showed that recipients with no history of hypertension reported the vaccine-related symptoms including neurological, systematic, gastrointestinal, respiratory, and local manifestations, significantly further than participants with high blood pressure with a p-value of 0.019, 0.004, 0.026, 0.015, and 0.001, respectively. Additionally, individuals with diabetes showed fewer symptoms than recipients without diabetes, but only systematic symptom incidence was statistically significant (p-value = 0.026). Moreover, gastrointestinal manifestations were significantly less reported in recipients with cardiac diseases, with a p-value of 0.039 compared to persons with no history of cardiac disorders. The details of the results are presented in **Table 3**.

**Table 3:** The occurrence of adverse events after receiving the first and second dose in the participants with underlying chronic disease.

	Variable	Category	First / second dose symptoms									
			Neurological N (%)		Systematic N (%)		Gastrointestinal N (%)		Respiratory N (%)		Local N (%)	
			First	second	First	second	First	second	First	second	First	second
**Underlying diseases**	**Cardiac**	**Yes**	13 (5.6)	6 (10.5)	22 (6.1)	11 (8.1)	3 (2.7)	1 (4.8)	0 (0)	1 (10)	11 (5.2)	8 (11.9)
		**No**	219 (94.4)	51 (89.5)	339 (93.9)	125 (91.9)	108 (97.3)	20 (95.2)	32 (100)	9 (90)	200 (94.8)	59 (88.1)
		**P value**	0.214	0.703	0.111	0.550	**0.039**	0.466	0.106	0.932	0.151	0.383
	**Lung diseases**	**Yes**	8 (3.4)	5 (8.8)	10 (2.8)	6 (4.4)	3 (2.7)	2 (9.5)	2 (6.2)	3 (30)	5 (2.4)	2 (3)
		**No**	224 (96.6)	52 (91.2)	350 (97.2)	130 (95.6)	108 (97.3)	19 (90.5)	30 (93.8)	7 (70)	206 (97.6)	65 (97)
		**P value**	0.281	**0.017**	0.748	0.444	0.971	0.120	0.189	**0.000**	0.724	0.795
	**Diabetes**	**Yes**	14 (6)	9 (15.8)	25 (6.9)	18 (13.2)	8 (7.2)	1 (4.8)	0 (0)	1 (10)	18 (8.5)	8 (11.9)
		**No**	218 (94)	48 (84.2)	336 (93.1)	118 (86.8)	103 (92.8)	20 (95.2)	32 (100)	9 (90)	193 (91.5)	59 (88.1)
		**P value**	0.064	0.253	**0.026**	0.380	0.605	0.320	0.076	0.883	0.919	0.889
	**Blood pressure**	**Yes**	25 (10.8)	9 (15.8)	44 (12.2)	22 (16.2)	9 (8.1)	0 (0)	0 (0)	0 (0)	18 (8.5)	13 (19.4)
		**No**	207 (89.2)	48 (84.2)	317 (87.8)	114 (83.8)	102 (91.9)	21 (100)	32 (100)	10 (100)	193 (91.5)	54 (80.6)
		**P value**	**0.019**	0.489	**0.004**	0.258	**0.026**	**0.021**	**0.015**	0.118	**0.001**	0.933
	**Hepatic**	**Yes**	3 (1.3)	3 (5.3)	6 (1.7)	3 (2.2)	1 (0.9)	0 (0)	1 (3.1)	0 (0)	3 (1.4)	0 (0)
		**No**	229 (98.7)	54 (94.7)	354 (98.3)	133 (97.8)	110 (99.1)	21 (100)	31 (96.9)	10(100)	208 (98.6)	67 (100)
		**P value**	0.948	**0.041**	0.213	0.739	0.651	0.508	0.357	0.682	0.870	0.198
	**Renal**	**Yes**	2 (0.9)	0 (0)	3 (0.8)	0 (0)	2 (1.8)	0 (0)	0 (0)	0 (0)	2 (0.9)	0 (0)
		**No**	230 (99.1)	57 (100)	357 (99.2)	136 (100)	109 (98.2)	21 (100)	32 (100)	10 (100)	209 (99.1)	67 (100)
		**P value**	0.957	0.412	0.817	0.128	0.235	0.641	0.579	0.752	0.894	0.356
	**Cancer**	**Yes**	3 (1.3)	1 (1.8)	4 (1.1)	2 (1.5)	2 (1.8)	1 (4.8)	0 (0)	1 (10)	0 (0)	0 (0)
		**No**	229 (98.7)	56 (98.2)	356 (98.9)	134 (98.5)	109 (98.2)	20 (95.2)	32 (100)	9 (90)	211 (100)	67 (100)
		**P value**	0.341	0.241	0.310	0.105	0.235	**0.014**	0.579	**0.000**	0.060	0.460
	**others**	**Yes**	23 (9.9)	3 (5.3)	36 (10)	14 (10.3)	8 (7.2)	1 (4.8)	1 (3.1)	0 (0)	15 (7.1)	8 (11.9)
		**No**	209 (90.1)	54 (94.7)	325 (90)	122 (89.7)	103 (92.8)	20 (95.2)	31 (96.9)	10 (100)	196 (92.9)	59 (88.1)
		**P value**	0.629	0.093	0.393	0.485	0.182	0.304	0.156	0.240	0.024	0.956

### 3.4 The prevalence of side effects among participants with the underlying chronic disease after the second dose of AstraZeneca COVID-19 vaccine

The existence of chronic diseases in both dose recipients who showed side effects was evaluated. Findings revealed that the incidence of neurological and respiratory symptoms in the participants with chronic lung disease was significantly fewer than in healthy persons, with a p-value of 0.017 and 0.000, respectively. Furthermore, the recipients with hypertension were shown all the vaccine-related adverse effects, including neurological, systematic, gastrointestinal, respiratory, and local symptoms, fewer than participants without hypertension. Albeit, only gastrointestinal manifestation was statistically significant (p-value = 0.021). Based on findings, neurological symptoms were less in recipients with hepatic than in individuals with no history of hepatic (p-value = 0.041). Additionally, recipients with cancer reported significantly fewer complications about gastrointestinal and respiratory symptoms compared to non-cancer participants (p-value = 0.014 and 0.000). Detailed information is provided in **Table 3**.

### 3.5. The prevalence of side effects among participants in terms of demographic characteristics after the first dose of AstraZeneca COVID-19 vaccine

The correlation between vaccine-related adverse effects after receiving the first dose and the demographic characteristics of participants were analyzed. In general, the demographic characteristics, including the age of 18–34 (p-value = 0.000), gender as female (p-value = 0.013), and smoking (p-value = 0.001 can influence the occurrence of post-vaccination adverse events after the first dose receiving (**S2 Table**). Findings showed that all of the side effects, including neurological, systematic, gastrointestinal, respiratory, and local symptoms, presented in the most frequency in the age group 18–34 in comparison to other ages significantly with the p-value of <0.0001, <0.0001, <0.0001, <0.0001, and <0.0001 respectively. The evaluation of post-vaccination side effects incidence between genders revealed that females reported all of the mentioned adverse effects more than males. Hence, neurological, systematic, gastrointestinal, and local symptoms significantly with a p-value of 0.020, 0.004, 0.001, and 0.000, respectively, and respiratory symptoms non-significantly were most common in females. Furthermore, results showed that non-smoker recipients reported more systematic symptoms (p-value = 0.001) than smokers. There was no significant relation between the other demographic data of vaccine recipients and the presence of side effects. Based on the current study, a significant relationship between the history of COVID-19 and the occurrence of each symptom after the first dose injection was not seen. Complementary information is shown in **Table 4.**

**Table 4:** The prevalence of side effects among participants in terms of demographic characteristics after first and second dose of AstraZeneca COVID-19 vaccine.

Variable	Category	First / second dose symptoms									
		Neurological N (%)		Systematic N (%)		Gastrointestinal N (%)		Respiratory N (%)		Local N (%)	
		First	second	First	second	First	second	First	second	First	second
**Age**	**18–34**	149 (64.2)	32 (56.2)	220 (60.9)	67 (49.3)	82 (73.9)	15 (71.4)	28 (87.5)	9 (90)	147 (69.7)	37 (55.2)
	**35–65**	64 (27.6)	21 (36.8)	101 (28)	53 (39)	27 (24.3)	6 (28.6)	4 (12.5)	1 (10)	58 (27.5)	26 (38.8)
	**≥65**	19 (8.2)	4 (7)	40 (11.1)	16 (11.8)	2 (1.8)	0 (0)	0 (0)	0 (0)	6 (2.8)	4 (6)
	**P value**	**<0.0001**	**0.001**	**<0.0001**	**<0.0001**	**<0.0001**	**0.005**	**<0.0001**	**0.009**	**<0.0001**	**<0.0001**
**Gender**	**Male**	108 (46.6)	25 (43.9)	175 (48.5)	65 (47.8)	43 (38.7)	10 (47.6)	13 (40.6)	4 (40)	85 (40.3)	34 (50.7)
	**Female**	124 (53.4)	32 (56.1)	186 (51.5)	71 (52.2)	68 (61.3)	11 (52.4)	19 (59.4)	6 (60)	126 (59.7)	33 (49.3)
	**P value**	**0.020**	0.072	**0.004**	**0.034**	**0.001**	0.506	0.186	0.340	**0.000**	0.475
**Blood type**	**A**	75 (32.3)	13 (22.8)	123 (34.1)	42 (30.9)	33 (29.7)	4 (19)	10 (31.3)	1 (10)	70 (33.2)	20 (29.9)
	**B**	38 (16.4)	11 (19.3)	61 (16.9)	23 (16.9)	20 (18)	9 (42.9)	5 (15.6)	5 (50)	35 (16.6)	11 (16.4)
	**O**	86 (37.1)	22 (38.6)	128 (35.5)	52 (38.2)	42 (37.8)	3 (14.3)	15 (46.8)	3 (30)	76 (36)	25 (37.3)
	**AB**	33 (14.2)	11 (19.3)	49 (13.5)	19 (14)	16 (14.5)	5 (23.8)	2 (6.3)	1 (10)	30 (14.2)	11 (16.4)
	**P value**	0.798	0.386	0.674	0.852	0.776	**0.005**	0.369	0.056	0.971	0.920
BMI	**Underweight**	12 (5.1)	2 (3.5)	13 (3.6)	2 (1.5)	7 (6.4)	1 (4.8)	2 (6.3)	0 (0)	8 (3.8)	1 (1.5)
	**Normal**	105 (45.3)	15 (26.3)	150 (41.7)	45 (33.1)	53 (47.7)	9 (42.8)	21 (65.6)	6 (60)	93 (44.1)	19 (28.4)
	**Overweight**	89 (38.4)	27 (47.4)	144 (40)	63 (46.3)	40 (36)	10 (47.6)	8 (25)	3 (30)	79 (37.4)	35 (52.2)
	**Obese**	26 (11.2)	13 (22.8)	53 (14.7)	26 (19.1)	11 (9.9)	1 (4.8)	1 (3.1)	1 (10)	31 (14.7)	12 (17.9)
	**P value**	**0.018**	0.419	0.639	0.379	0.064	0.368	**0.012**	0.431	0.601	0.264
Smoke	**0**	206 (88.8)	48 (84.2)	320 (88.6)	114 (83.8)	99 (89.2)	19 (90.5)	32 (100)	9 (90)	187 (88.6)	54 (80.6)
	**1–10 (smoker)**	15 (6.5)	3 (5.3)	24 (6.6)	10 (7.4)	8 (7.2)	0 (0)	0 (0)	0 (0)	13 (6.2)	7 (10.4)
	**>10 (heavy smoker)**	11 (4.7)	6 (10.5)	17 (4.7)	12 (8.8)	4 (3.6)	2 (9.5)	0 (0)	1 (10)	11 (5.2)	6 (9)
	**P value**	0.106	0.116	**0.001**	0.062	0.596	0.237	0.065	0.500	0.085	0.418
COVID-19 history	**Yes**	96 (41.4)	30 (52.6)	152 (42.1)	69 (50.7)	52 (46.8)	13 (61.9)	13 (40.6)	5 (50)	89 (42.2)	28 (41.8)
	**No**	136 (58.6)	27 (47.4)	209 (57.9)	67 (49.3)	59 (53.2)	8 (38.1)	19 (59.4)	5 (50)	122 (57.8)	39 (58.2)
	**P value**	0.526	**0.042**	0.064	**0.001**	0.088	**0.040**	0.936	0.532	0.367	0.828
**Disease severity**	**ICU**	4 (4.2)	4 (12.1)	5 (3.3)	4 (5.3)	3 (6.5)	1 (7.7)	0 (0)	1 (20)	2 (2.4)	1 (3)
	**Non-ICU**	92 (95.8)	29 (87.9)	148 (96.7)	72 (94.7)	43 (93.5)	12 (92.3)	13 (100)	4 (80)	83 (97.6)	32 (97)
	**P value**	0.221	**<0.0001**	0.332	0.069	0.072	0.283	0.528	**0.021**	0.752	0.965

### 3.6 The prevalence of side effects among participants in terms of demographic characteristics after the second dose of AstraZeneca COVID-19 vaccine

The relation between the post-vaccination adverse events occurrence and demographic characteristics of recipients was evaluated. Results showed that the demographic characteristics, including age (p-value = 0.000), gender (p-value = 0.044), history of SARS-CoV-2 infection (p-value = 0.004), and COVID-19 severity (p-value = 0.000), play a role in the presence of vaccine-related adverse events after receiving the second dose (**S2 Table**). The age group 18–34 reported all symptoms in increasing numbers compared to other ages. Based on the statistical analysis, the presence of neurological (56.2%), systematic (49.3%), gastrointestinal (71.4%), respiratory (90%), and local symptoms (55.2%) were significantly frequent in recipients in 18–34 years old with the p-value of 0.001, <0.0001, 0.005, 0.009, <0.0001 respectively. The evaluation of gender dependency in the presence of side effects revealed that most of the adverse symptoms, including neurological, systematic, gastrointestinal, and respiratory, but not local manifestations, were highly reported in females compared to males; however, only systematic symptoms were statistically significant (p-value = 0.034). Moreover, gastrointestinal manifestation was significantly prevalent in recipients with B blood type after the second dose injection (p-value = 0.005). Furthermore, the findings indicated that recipients with a history of COVID-19 infection had a higher incidence of neurological, systematic, and gastrointestinal symptoms (p-value = 0.042, 0.001, 0.040, respectively) but the total number of reported adverse effects didn’t follow this pattern. Moreover, the patients who did not require the intensive care medicine at Intensive Care Unit, (non-ICU group) based on disease severity, reported significantly more side effects, including neurological (p-value = <0.0001) and respiratory (p-value = 0.021). More detailed information can be found in **Table 4.**

### 3.7 Correlation between BMI and side effects after AstraZeneca COVID-19 vaccine

Body Mass Index of all participants was calculated, and its relation with the reported reactions, including neurological, systematic, gastrointestinal, respiratory, and local symptoms after receiving the first and both doses, was evaluated. According to our findings, the BMI of the recipients can influence the frequency of respiratory reactions. Hence, respiratory manifestation was reported frequently after receiving the first dose in an individual with a mean BMI of 22.61 ± 3.02 compared with non-reacted recipients (24.34 ± 4.47) with a p-value of 0.028. However, a significant relationship between the presence of other side effects after vaccination and the BMI of the participants was not found.

### 3.8 Factors associated with experiencing side-effects after the first and second dose of Oxford/AstraZeneca vaccine

We categorize all symptoms into local and systemic side effect and then association between underlying disease and side effects were analyzed. Biased on our result, underlying disease is associated with the occurrence of systemic complications in the first dose of the vaccine, (OR = 0.5, 95% CI 0.3,0.82, p value = 0.04) and also having underlying diseases increases the rate of experiencing local complications after the first dose of the vaccine. (OR = 0.49, 95% CI 0.32,0.74, p value = 0.01). However, there was no significant association between underlying diseases and the occurrence of systemic and local side effects after the second dose. The association of the recipient’s demographic characteristics and the probability the post-vaccination adverse events occurrence was analyzed with the logistic regression. Results showed that increasing age leads to a significant reduction in post-vaccination adverse reactions following receiving the first dose (p-value = 0.000) and both dose (p-value = 0.000). as regards the age of the vaccine recipients may be a predictive factor in the occurrence of vaccine-related side effects. Complementary information is listed respectively in **Table 5**.

**Table 5:** Regression model for predicting general side effects in first or second dose of AstraZeneca vaccine.

Variable	B	S.E	P value	Exp(B)	95% C.I.for EXP(B)	
					Lower	Upper
**Age**	-0.079	0.017	**0.000**	0.924	0.894	0.956
**Gender**	-0.063	0.577	0.913	0.939	0.303	2.908
**BMI**	-0.028	0.060	0.635	0.972	0.865	1.092
**Smoke**	-0.087	0.513	0.864	0.916	0.336	2.502
**Blood type**	-0.198	0.213	0.353	0.820	0.540	1.246
**Underlying diseases**	0.852	0.688	0.215	2.345	0.609	9.023
**Covid history**	0.133	0.783	0.865	1.142	0.246	5.298
**Disease severity**	0.534	0.724	0.460	1.706	0.413	7.049

*Age: 1–18–34, 2–35–64, 3- ≥65 *Gender: 1- Male, 2- Female *BMI: 1-Underweight, 2-Normal, 3-Overweight, 4- Obese * Smoke: 0, 1-10(smoker), >10(heavy smoker) *Blood type: 1-A, 2-B, 3-O, 4-AB *Underlying diseases: 1-Yes, 2-No *Covid history: 1-Yes, 2-No *Disease severity: 1-ICU, 2-Non-ICU

## 4. Discussion

However, the vaccine is the most effective tool to fight against most viral pandemics. Still, several factors, such as vaccine safety, effectiveness, complications, and side effects, may influence in acceptance or rejection of vaccination [20, 21]. Among the various types of vaccines against COVID-19, Oxford-AstraZeneca was a widely used viral vector-based vaccine and was the second most frequently administered vaccine in Iran [22]. In the current study, we aimed to evaluate post-vaccination adverse effects in ChAdOx1 nCoV-19 (Oxford-AstraZeneca) vaccine recipients. Findings showed that most post-vaccination adverse events were reported in about 1.24 days after the first dose and 1.83 days second dose injection and prolonged less than a week. All the side effects were temporary and disappeared without any need for hospitalization. Other studies reported that most side effects presented in the first two days of vaccination and continued for three days [10, 20]. Also, the duration of adverse effects was reported to be less than seven days [23]. A study in Saudi Arabia showed that 93.6% of the side effects after the Oxford-AstraZeneca vaccine injection were reported on the day of receiving the vaccine, most of which resolved in five days. Only 2.4% of post-vaccine side effects last over seven days [24]. Moreover, our finding was the same trend as other studies, which demonstrated that most Oxford-AstraZeneca vaccine-related side effects were mild to moderate in severity, tolerable, and self-limiting, with no need for hospitalization [15, 25, 26]. Our findings revealed that the number of reported adverse events after the first dose injection was more than the second. A study by Maha Farhat reported a high number of side effects after the first dose of the Oxford-AstraZeneca vaccine (91.7%) in comparison to the second dose (70.4%) [27]. Also, studies conducted in Iran documented that participants experienced a higher incidence of side effects after the first dose of the Oxford-AstraZeneca vaccine compared to the second dose.[22, 28]. This result complied with the phase 2/3 trial of ChAdOx1 nCoV-19, which reported fewer adverse events after the second dose injection than after the first [25]. However, some studies indicated that the vaccine-related side effects were more prevalent after the second dose than after the first dose [23, 29]. The exact reason is unknown, but this discrepancy may relate to sample size, demographic characteristics of vaccine recipients, and their willingness to inject the second dose.

In general, systematic symptoms were the most frequent side effect after receiving the first and the second dose in the current study. Among the systematic manifestation, body pain, fainting, and fever are the most prevalent reported adverse events after the first and the second dose injection. After that, injection site pain as a local symptom, and headaches were commonly reported. Despite some studies that reported the local injection site pain as the most reported side effect [24, 30–32]. several studies documented the systemic symptoms as the most frequent side effect in Oxford-AstraZeneca recipients [14, 33]. According to Kaura et al. and Farhat et al. studies, Oxford-AstraZeneca vaccine side effects presented systemic symptoms more than local ones [27]. Based on our results, adverse reactions in the injection site were reported at 77.1% after the first doses, thus congruence with the study in Ethiopia, which reported 75.8% [29]. However, it was higher than the assessments in the UK and the Kurdistan Region of Iraq [23, 34]. This variance may result from differences in examined population characteristics, race, sample size, and study design [35, 36]. Moreover, fainting was one of the frequently reported side effects following vaccination in the current survey. There wasn’t a similar assessment in other studies. However, it was noted that 42.3% of the respondents reported being hesitant and concerned about vaccine safety before receiving the vaccine [3, 20]. In this regard, if stress and fear before vaccination can lead to fainting after injection should be evaluated in future studies. The evaluation of demographic characteristics in the participants who reported post-vaccination side effects revealed that 18-34-year-old recipients presented more adverse reactions than the elderly. Logistic regression analysis of the relationship between post-vaccination side effects and the recipient’s age showed a significant negative correlation. In other words, increasing age led to a decrease in adverse events after the first and second dose injection. This was not a surprising finding, and several studies considered that the side effects were more prevalent in young individuals [23, 37–39]. Also, these findings were consistent with the results of Oxford AstraZeneca’s clinical trial [25]. A strong immune reaction in young people under 50 due to robust inflammatory cytokines production may explain this high frequency of side effects in younger recipients [40]. Furthermore, we found that females presented significantly more adverse events following the first dose and second dose vaccination than males. However, this pattern was not reported in the Oxford/AstraZeneca clinical trials, but previous studies showed similar findings. A study in Bangladesh revealed that females were 1.5 times more prone to complain of adverse reactions after receiving the Oxford AstraZeneca vaccine than males [39]. Likewise, recent studies in Iran and Saudi Arabia indicated that females reported a higher number of symptoms than males following the Oxford/AstraZeneca injection [22, 41]. Several factors can explain this gender dependency in the incidence of vaccine side effects. The influence of hormones on immune responses might be associated with this variation in males and females [42, 43]. In the current study, the non-smoker participants were more prone to present post-vaccination side effects. Hence, adverse symptoms, especially systemic reactions, were significantly high in recipients who didn’t consume nicotine. The study by M. Hatmal reported that non-smokers had more chance to present injection site pain and swelling after COVID-19 vaccine administration [44]. Also, the study in Saudi Arabia documented the high frequency of side effects in non-smoking recipients [45]. Additionally, it was noted that the Oxford/AstraZeneca recipients who did not consume nicotine were more susceptible to feeling adverse effects after vaccination [46]. Despite contrary results which reported smoking as a risk factor for incidence of post-vaccination side effects [47], there was evidence of the disruptive influence of smoking on the immune system and antibody responses [48]. In this regard, Watanabe et al. conducted a study that revealed lower serum antibody titer concentrations in smokers who received the Pfizer COVID-19 vaccine [49]. Accordingly, it might be the reason for weak adverse reactions in smoker participants. Based on statistical analysis, the total number of reported side effects was significantly high in participants without a previous history of COVID-19. However, logistic regression analysis did not show a significant association between the recipient’s history of COVID-19 infection and the risk of side effect presence following vaccination. Similar to our findings, an increasing number of side effects were reported in recipients without previous SARS-CoV-2 infection [50, 51]. There were contradictory reports regarding the increased chance of post-vaccination side effects in recipients with a prior history of COVID-19[23, 52]. The main reason for this event is unknown but it is possible that individuals who have not had COVID-19 before may have a strong immune response after receiving the vaccine for the first time, resulting in additional side effects [53]. These conflicting findings could be attributed to the difference in the number of participants with previous COVID-19 who were willing to receive one or both vaccine doses. The logistic regression analysis of COVID-19 severity and post-vaccination side effects showed a significant negative correlation. The previous history of non-severe COVID-19 was found to be a predisposing factor for adverse events occurring after the second dose. These findings may be related to a smaller number of individuals who survived a severe COVID-19 infection and then received the vaccine. Further studies with large sample sizes are required to illustrate the role of COVID-19 history in post-vaccination reactions. Also, based on statistical analysis, the total number of reported side effects was significantly high in participants without previous history of COVID-19. Similar to our findings, numerous side effects in the recipients without previous SARS-CoV-2 infection were reported [17, 54]. People with no prior experience of COVID-19 may induce a robust immune response after the vaccine injection for the first time, consequently causing extra side effects [53]. However, contradictory reports showed that recipients with prior history of COVID-19 had an increased chance of post-vaccination side effects [23]. These conflicting findings could be attributed to the difference in the number of participants with previous COVID-19 who had been willing to receive a single or both vaccine doses. Our findings showed that participants with normal BMI (>25 kg/m2) reported more adverse events after the first dose injection. In addition, the mean BMI of the recipients who reported side effects was lower than those who didn’t have adverse symptoms. Likewise, in the study of Ibrahim Amer et al., COVID-19 vaccine recipients with normal BMI were more prone to present side effects [45]. On the other hand, similar to our findings, a significant association between high BMI and post-vaccine side effects was not reported [47]. In general, there wasn’t a significant correlation between the ABO blood type of participants and the occurrence of post-vaccination side effects in the current survey [51, 55].

The evaluation of underlying disease in participants was performed. Results showed that the recipients who suffer from chronic disorders, including hypertension, diabetes, cancer, and pulmonary disease, reported fewer side effects after vaccination. Contrary to our findings, some studies considered comorbid diseases as a risk factor for adverse effects present post-vaccination [22, 56]. On the other hand, any significant influence of cancer, receiving chemotherapy or having a comorbidity on the risk of developing adverse events after vaccination was rejected. [50, 57–60]. However, the reduced immune response to the vaccine in recipients with chronic respiratory disorder [61–64], chronic liver disease [65], hypertension [47, 49], and cancer [50, 66, 67] were demonstrated in several studies [68, 69]. Moreover, the myelosuppressive effect of chemotherapy was seen in cancer patients, which reduced vaccine immunogenicity and reactogenicity [70, 71]. Also, overlapping the post-vaccination adverse events with the symptoms of chronic disease and therapies, plus the higher tolerance of patients due to their chronic illness, may explain the fewer vaccine-related side effects in recipients with chronic disease [50].

Sampling was identified as a limitation in this study. All participants in our study were solely from Ardabil, a small province in Iran, and they may not have been a representative sample of the vaccinated population. Another limitation was the failure to assess the severity of adverse effects. Also, time constraints prevented us from following up with individuals who had received the vaccine, so our assessment was limited to short-term side effects only. To investigate delayed adverse effects, follow-up studies would be necessary. Another limitation is failing to provide COVID-19 occurrence rates post vaccination and that asymptomatic COVID-19 cannot be fully excluded.

One of the strengths of this study was the balance in the number of participants according to their gender. The total number of females and males was nearly equal, making the comparison of the side effects between the two genders statistically reliable. Also, there were no monetary or other motivations for participation in this study, and all the individuals were aware of anonymity and voluntary participation. In addition, there were no restrictions based on occupation or economic status for volunteers and all citizens of Ardabil province, Iran, who received the first or both doses of the ChAdOx1-nCoV-19 were included in the study.

## 5. Conclusion

The ChAdOx1 nCoV-19 (Oxford-AstraZeneca) vaccine has been widely administered and has shown promising protective effects against COVID-19. However, adverse reactions after vaccination are inevitable. Studies have also identified risk factors such as gender and age that make certain individuals more susceptible to experiencing these adverse effects. Moreover, having underlying diseases could increase the rate of experiencing local complications after the first dose of the vaccine. In general, it is important to note that, these side effects are not life-threatening and most of them are mild to moderate typically resolving on their own within a few days.

## Supporting information

S1 Table The occurrence of adverse events after receiving the first and second dose in the participants with underlying chronic disease. (DOCX)

S2 Table The prevalence of side effects among participants in terms of demographic characteristics after first and second dose of AstraZeneca COVID-19 vaccine. (DOCX)

## Abbreviations

BMI: Body Mass Index
CDC: Centers for Disease Control and Prevention
COVID-19: Coronavirus disease 2019
ICU: Intensive Care Unit
SARS-CoV-2: Severe acute respiratory syndrome coronavirus 2
VAERS: Vaccine Adverse Event Reporting System
