# Supplementary material for: Reported side-effects following Oxford/AstraZeneca COVID-19 vaccine in the north-west province, Iran: A cross-sectional study
Source: PLoS One. 2024 Jan 5;19(1):e0296669. doi: 10.1371/journal.pone.0296669 (PMC10769020; doi:10.1371/journal.pone.0296669)
Supplement: S1 Table — (DOCX) [file pone.0296669.s001.docx]

**S1 Table. The occurrence of adverse events after receiving the first and second dose in the participants with underlying chronic disease**

| Variable | Category | First dose symptoms | | Second dose symptoms | |
| --- | --- | --- | --- | --- | --- |
|  |  | **N (%)** | **P value** | **N (%)** | **P value** |
| Cardiac | **Yes** | 25 (6.6) | 0.337 | 15 (9.3) | 0.945 |
|  | **No** | 356 (93.4) |  | 146 (90.7) |  |
| Respiratory | **Yes** | 10 (2.6) | 0.944 | 6 (3.7) | 0.825 |
|  | **No** | 370 (97.4) |  | 155 (96.3) |  |
| Diabetes | **Yes** | 29 (7.6) | 0.170 | 20 (12.4) | 0.571 |
|  | **No** | 352 (92.4) |  | 141 (87.6) |  |
| Blood pressure | **Yes** | 48 (12.6) | **0.006** | 25 (15.5) | 0.104 |
|  | **No** | 333 (87.4) |  | 136 (84.5) |  |
| Hepatic | **Yes** | 6 (1.6) | 0.283 | 3 (1.9) | 0.950 |
|  | **No** | 374 (98.4) |  | 158 (98.1) |  |
| Renal | **Yes** | 4 (1.1) | 0.382 | 0 (0) | 0.074 |
|  | **No** | 376 (98.9) |  | 161 (100) |  |
| Cancer | **Yes** | 4 (1.1) | 0.382 | 2 (1.2) | 0.167 |
|  | **No** | 376 (98.9) |  | 159 (98.8) |  |
| others | **Yes** | 37 (9.7) | 0.159 | 18 (11.2) | 0.750 |
|  | **No** | 344 (90.3) |  | 143 (88.8) |  |
