# Supplementary material for: Reported side-effects following Oxford/AstraZeneca COVID-19 vaccine in the north-west province, Iran: A cross-sectional study
Source: PLoS One. 2024 Jan 5;19(1):e0296669. doi: 10.1371/journal.pone.0296669 (PMC10769020; doi:10.1371/journal.pone.0296669)
Supplement: S2 Table — (DOCX) [file pone.0296669.s002.docx]

| Variable | Category | First dose symptoms | | Second dose symptoms | |
| --- | --- | --- | --- | --- | --- |
|  |  | **N (%)** | **P value** | **N (%)** | **P value** |
| Age | **18-34** | 228 (59.8) | **0.000** | 83 (51.6) | **0.000** |
|  | **35-65** | 105 (27.6) |  | 57 (35.4) |  |
|  | **≥65** | 48 (12.6) |  | 21 (13.0) |  |
| Gender | **Male** | 188 (49.3) | **0.013** | 79 (49.1) | **0.044** |
|  | **Female** | 193 (50.7) |  | 82 (50.9) |  |
| Blood type | **A** | 130 (34.1) | 0.787 | 52 (32.3) | 0.798 |
|  | **B** | 62 (16.3) |  | 26 (16.1) |  |
|  | **O** | 134 (35.2) |  | 60 (37.3) |  |
|  | **AB** | 55 (14.4) |  | 23 (14.3) |  |
| BMI | **Underweight** | 14 (3.7) | 0.781 | 3 (1.9) | 0.430 |
|  | **Normal** | 156 (41.1) |  | 54 (33.5) |  |
|  | **Overweight** | 153 (40.3) |  | 74 (46.0) |  |
|  | **Obese** | 57 (15.0) |  | 30 (18.6) |  |
| Smoke | **0** | 336 (88.2) | **0.001** | 136 (84.5) | 0.077 |
|  | **1-10** (smoker) | 27 (7.1) |  | 12 (7.5) |  |
|  | **>10**  (heavy smoker) | 18 (4.7) |  | 13 (8.1) |  |
| COVID-19 history | **Yes** | 160 (42.0) | **0.042** | 78 (48.4) | **0.004** |
|  | **No** | 221 (58.0) |  | 83 (51.6) |  |
| Disease severity | **ICU** | 5 (2.9) | 0.069 | 4 (4.4) | **0.000** |
|  | **Non-ICU** | 170 (97.1) |  | 87 (95.6) |  |

**S2 Table.** **The prevalence of side effects among participants in terms of demographic characteristics after first and second dose of AstraZeneca COVID-19 vaccine**
